# NLRC3: A Novel Noninvasive Biomarker for Pulmonary Hypertension Diagnosis

**DOI:** 10.14336/AD.2017.1102

**Published:** 2018-10-01

**Authors:** Li-huang Zha, Jun Zhou, Tang-zhiming Li, Hui Luo, Jing-ni He, Lin Zhao, Zai-xin Yu

**Affiliations:** ^1^Department of Cardiology, Xiangya Hospital, Central South University, Changsha, Hunan, China; ^2^Medical Science Research Center, Xiangya Hospital, Central South University, Changsha, Hunan, China; ^3^Department of Cardiology, Zhuzhou Central Hospital, Zhuzhou, Hunan, China

**Keywords:** pulmonary hypertension, right heart catheterization, NLRC3

## Abstract

The nucleotide-oligomerization domain (NOD)-like receptor subfamily C3 (NLRC3) is a newly discovered and incompletely characterized member of the NLR family which negatively regulates inflammatory responses. Inflammation is considered a critical pathogenesis in pulmonary hypertension (PH). This is the first study to hypothesize that NLRC3 is closely correlated with PH. Total of 43 PH patients who were diagnosed by right heart catheterization (RHC) and 20 age-matched healthy control subjects were included. Echocardiographic variables and blood biochemical parameters were tested. Results of World Health Organization functional class (WHOFC), Borg dyspnea score and 6-minute walk tests (6MWT) were recorded. Mean pulmonary arterial pressure (mPAP) and pulmonary vascular resistance (PVR) were measured from RHC. Serum NLRC3 concentrations were detected by ELISA. ROC curve analysis was used to evaluate the diagnostic value of NLRC3 concentrations in PH. We found that serum NLRC3 concentration was significantly decreased in PH compared to the healthy control group. Serum NLRC3 concentration correlated negatively with mPAP and PVR. In addition, a negative correlation between serum NLRC3 concentration and WHOFC were detected. We proposed a cut-off value of 2.897ng/mL for serum NLRC3 concentration which was able to predict PH with 88% sensitivity and 85% specificity. In conclusion, NLRC3 concentrations in PH were significantly decreased, suggesting that NLRC3 may potentially be a diagnosis index and represent a prognostic factor for PH patients.

Pulmonary hypertension (PH) is a progressive disease which leads to elevation of pulmonary arterial pressure, pulmonary vascular resistance and right ventricular failure. The pathogenesis of pulmonary hypertension is not completely known. It is considered to be a multifactorial pathophysiology. Inflammation and immunity play an important role in its occurrence and development, which can lead to abnormal cell proliferation and apoptosis [1]. Although novel therapies have improved survival to a certain extent, the prognosis of PH is still poorly maintained [2]. Considering the high mortality and disability rates, providing timely diagnosis and accurate treatment are essential for improving the prognosis for PH patients.

The current commonly used diagnostic methods are echocardiography, chest X-ray, electrocardiogram, cardiovascular magnetic resonance (CMR) and right heart catheterization (RHC). RHC is an invasive treatment and is well-known as the gold standard for PH diagnosis [3, 4]. It can accurately measure systolic/diastolic/mean pulmonary artery pressure (PAP), pulmonary capillary wedge pressure (PCWP), cardiac output (CO) and other parameters. Pulmonary vascular resistance (PVR) and cardiac index (CI) are calculated based on these measurements above[5]. PVR, right atrial pressure (RAP) and CI are all important indicators to assess the severity of PH. However, as an invasive examination, not only it is an expensive surgical diagnosis, it is limited by medical resource and may deliver potential complications to patients [6, 7].

Noninvasive screenings and monitoring for PH are challenging because of different limitations. Imaging techniques are widely utilized during the assessment, treatment, and observation in PH patients. Echocardiography is the most widely used method for detecting pulmonary artery pressure in many ways, but it always confined by the poor acoustic window. Right ventricular systolic pressure (RVSP) values can be gained by measuring the tricuspid regurgitant jet velocity and using the modified Bernoulli formula. When an estimated right atrial pressure adds to RVSP, this number is considered to equal the systolic PAP in the absence of pulmonic stenosis [8]. Several studies indicated that RVSP and sPAP obtained by echocardiography were only moderately correlated with RHC read-out values, and the diagnostic accuracy of echocardiography for PH is also modest [8-11]. CMR is not routinely available and may not be tolerable to patients with severe PH [12].

The WHO-FC is commonly used as a predictor of survival in PH, but the inter-observer variation is unstable among the evaluations [13, 14]. The 6-minute walk test (6MWT) is an objective assessment widely used to assess the efficacy of therapy in clinical trials. Borg dyspnea score (BDS) is a self-administered measurement tool that investigates breathlessness under exertion. BDS is often identified as part of the 6MWT in PH and often supposed as a secondary endpoint in clinical trials [15]. Many factors such as gait speed, age, weight and even encouragement from others can influence 6MWT and BDS values and lead to large variations [16].

Search for a simple non-invasive option is ongoing and several biomarkers are being studied. N-terminal pro-brain natriuretic peptide (NT-proBNP), vascular endothelial growth factor (VEGF), Galectin-3 and other serum proteins are considered associated with PH. However, none of these indexes can evaluate PH with high specificity and sensitivity, nor can they exhibit significant correlation with the progression of PH [3, 17-20]. Thus, looking for new approaches to evaluate PH has an important signification.

Nucleotide-binding oligomerization domain-like receptors (NLRs) are a family of intracellular proteins that play an important role in inflammation and immunity [21, 22]. NLR Family CARD Domain Containing 3 (NLRC3, also known as CLR16.2 or NOD3) is an incompletely characterized member of the NLR family. Some researchers recently found that NLRC3 can inhibit cell proliferation and inflammation and thus has the profile in cell antiproliferation and promoting proapoptotic signals [23]. We hypothesized that the level of serum NLRC3 in PH patients may be different from healthy people and that an evaluation of serum NLRC3 concentration may provide reliable noninvasive screening for PH diagnosis. In our study, we examined the serum NLRC3 concentration and investigated whether non-invasive serum NLRC3 measurement was closely correlated with other indexes such as hemodynamic parameters acquired by RHC in PH patients. The goals of this study were to establish a correlation between changes of serum NLRC3 and severity in PH disease, and to reveal the value of NLRC3 in the assessment of PH.

## MATERIALS AND METHODS

### Study subjects

This prospective study was performed in accordance with the ethical guidelines of the Declaration of Helsinki, and was approved by the Medical Ethics Committee of Xiangya Hospital of Central South University. Written informed consent was obtained from all subjects before enrollment. The study was performed between April 2012 and February 2017 at Xiangya Hospital, Changsha. We included patients with PH diagnosed by RHC (n=43) and healthy controls (n=20) matched for age and gender. Healthy controls were recruited during physical check-ups and were free of the conditions stated in the exclusion criteria. The diagnosis of PH was established by standard criteria. Inclusion criteria was characterized by an increase in a mean PA pressure > 25mmHg, as tested by RHC; exclusion criteria included acute or chronic bacterial and viral infections, sleep apnea, ischemic heart disease, diabetes, hypertension, cancer and liver disease.

#### Sample collection and measurement

Whole blood samples were collected through peripheral vein puncture in non-fasting subjects, processed to serum (immediate centrifugation for 20 minutes at 1300g), and stored at -80°C. Measurements of biochemical parameters, such as triglycerides and total cholesterol (TC), were performed fresh in the clinical laboratory at the hospital. Serum samples were stored at -80°C before being analyzed. All samples were thawed only once prior to use. Serum levels of NLRC3 were measured using commercially available enzyme-linked immunosorbent assay (ELISA) kits (Wuhan Abebio Science Co., Ltd. China; Catalogue #AE30906HU). According to the instructions, the intra-assay and inter-assay coefficients of variation were <8% and <12% for NLRC3.

### Data collection and definition

Baseline information was obtained at admission, including race, age, gender, history of health and medication use, smoking status, and alcohol use. Body mass index (BMI) was calculated as measured weight (kg) divided by the square of measured height (m^2^). Standardized WHOFC was provided by the treating physician in our hospital. 6MWT was performed according to guidelines.

### Echocardiography Studies

All examination procedures were performed to provide a comprehensive echocardiographic examination that would enable assessment of left ventricular (LV) and right ventricular (RV) systolic and diastolic function using several indexes. After acquiring two-dimensional images in the parasternal and apical views, pulsed-wave doppler was utilized to record transmitral and pulmonary venous (PV) flow in the apical four-chamber view. The sample volume size for acquiring mitral and PV flow signals was 1 to 2mm, and recordings were acquired for two to three respiratory cycles at a sweep speed of 100mm/s. Echocardiographic images were stored and analyzed off-line. Measurements of LV\RV end-diastolic dimension, ejection fraction (EF), and left\right atrium (LA\RV) maximum volume were performed per American Society of Echocardiography recommendations. Doppler measurements represent the average of three beats. Continuous-wave Doppler was utilized to record the tricuspid regurgitation (TR) jet from multiple windows. The systolic pulmonary artery pressure (sPAP) was derived using the modified Bernoulli equation (sPAP= 4v2+RA pressure, where v is the peak velocity of TR in meters per second), and an estimate of RA pressure using the diameter and collapse index of the inferior vena cava and the hepatic venous flow pattern. Mitral inflow was analyzed for peak early diastolic velocity (E), peak late diastolic velocity (A) and E/A ratio.

### Right Heart Catheterization

Medex transducers were balanced prior to the acquisition of the hemodynamic data, with the zero level at the midaxillary line. Pressure measurements were acquired from the right atrium, right ventricle, and pulmonary artery at end-expiration and represent the average of five cardiac cycles. PCWP was verified by chest radiograph and changes in pressure wave form. CO was determined by thermo dilution, in which three cardiac cycles with 10% variation were averaged. PVR was calculated.

### Statistics

Continuous variables with a normal distribution were expressed as mean±standard deviations; variables with a skewed distribution were expressed as medians (interquartile range). Categorical data were denoted as counts or percentages. Data analyses were performed using Statistical Package for the Social Sciences, version 18.0 (SPSS Inc., Chicago II, USA). Numeration data were statistically analyzed with Chi-square test. For comparison between two groups, we performed a Student’s t-test. A one-way analysis of variance (ANOVA) was used for analysis among three or more groups. A Pearson’s or Spearman’s correlation test and logistic regression analysis were applied to specify the relationships among the variables as indicated in the figure legend. ROC curve analysis was used to evaluate the diagnostic value and to define the diagnostic cut-off value of NLRC3 concentrations in PH. The range of suspicious value was also determined by ROC curve analysis. Values of p<0.05 were considered statistically significant.

**Table 1:** Baseline characteristics of the study population.

Characteristic	Patients (n=43)	Controls (n=20)	*p*-value
Age (years)	34.42 ± 12.59	39.92 ± 11.28	0.683
Male (%)	8 (19.5)	5(25.0)	0.341
BMI (kg/m^2^)	19.91 ± 2.35	20.96 ± 4.27	0.184
TG (mmol/L)	1.37 ± 0.69	1.28 ± 0.66	0.095
TC (mmol/L)	4.14 ± 0.94	4.36 ± 0.79	0.348
HDL-C (mmol/L)	1.21 ± 0.35	1.52 ± 0.33	0.466
LDL-C (mmol/L)	2.50 ± 0.82	2.37 ± 0.73	0.055

The continuous variables were expressed as the mean±standard deviation (SD) or the median (interquartile range). The categorical values were presented as the frequencies (percentages). No significant differences were found between the PH patients and controls in age, gender, body mass index (BMI), or cholesterol status. Abbreviations: BMI: body mass index; TG: triglycerides; TC: total cholesterol; HDL-C: high-density lipoprotein cholesterol; LDL-C: low-density lipoprotein cholesterol

**Figure 1.**
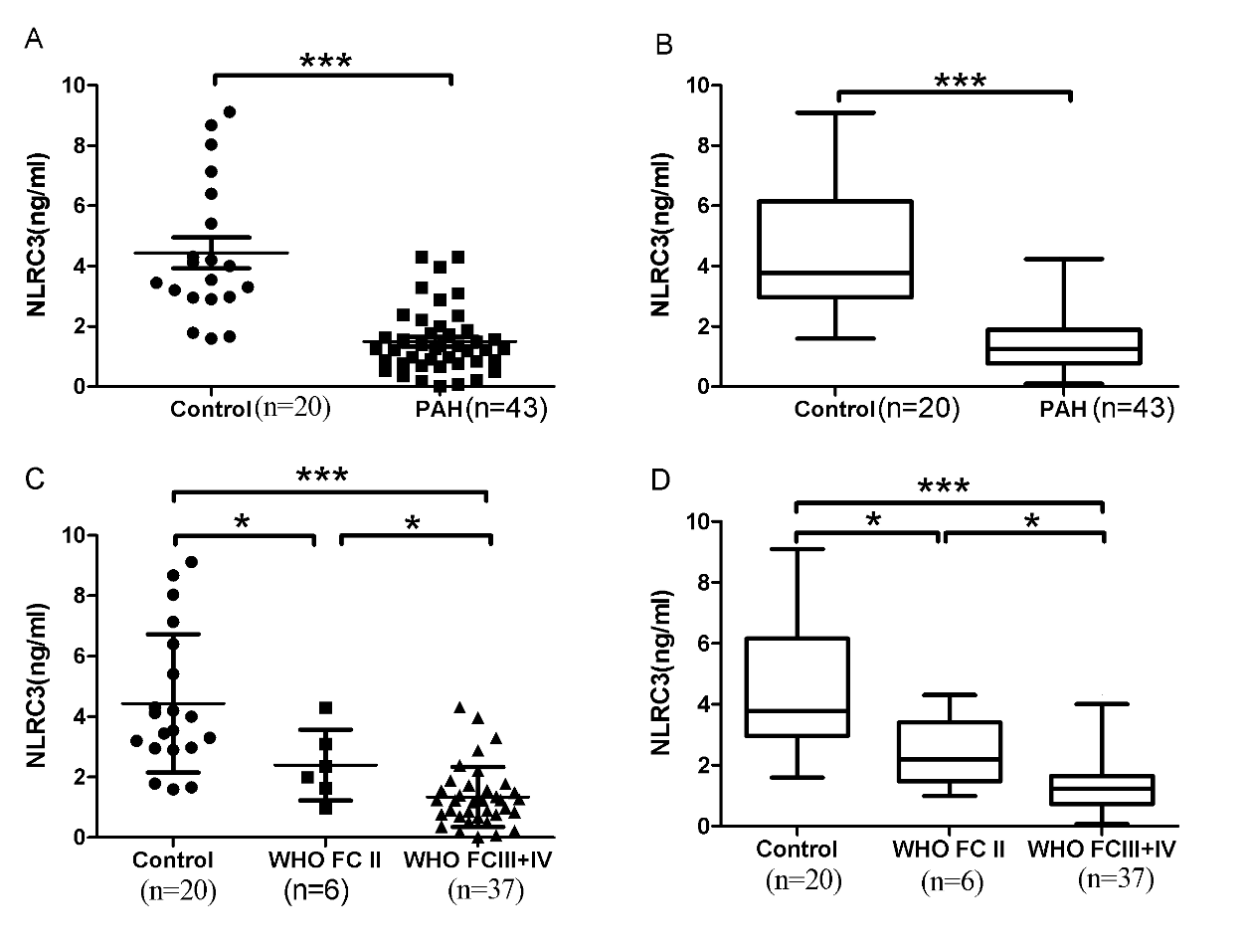
Serum NLRC3 concentrations were reduced in PH patients versus controls The scatter plots on the left and the Box-and-Whisker plots above on the right provided data for serum NLRC3 concentrations (Fig. 1A and 1B) in control subjects (n = 20), and patients with PH (n = 43). Serum NLRC3 concentrations decreased in PH severity. The scatter plots on the left and the Boxand-Whisker plots on the right below provided data for serum NLRC3 concentrations (Fig. 1C and 1D) according to WHO FC. Control: n=20; WHOFC II: n = 6; WHOFCIII-IV: n = 37. The scatter plots showed the mean±SD; the Box-and-Whisker plots showed the median with IQR±5-95th percentile. *p<0.05, ***p<0.0001.

## RESULTS

### Study participants background status were well-controlled

The study included 43 patients with PH and 20 age-and sex-matched health controls. All participants were of the Han nationality. The patients were between the ages of 18 to 75 years, with a mean age of 34.42 years, and a male-to-female ratio of 1:4. while the health controls ranged were between ages 20 to 76 years, with a mean age of 39.92 years, and a male-to-female ratio of 1:3. The BMI was 19.91±2.35 kg/m^2^ in PH patients versus 20.96±4.27 kg/m^2^ in controls. The value of triglycerides and cholesterols for all the subjects were shown in Table 1. No significant differences were found between the PH patients and controls in age, gender, body mass index (BMI), or cholesterol status (Table 1), an indication that both groups of participants were comparable to each other. Serum NLRC3 concentrations in PH patients were significantly lower than the control group, and was further decreased in WHOFC III-IV compared to WHOFC II in PH patients.

ELISAs were performed to evaluate if serum NLRC3 concentrations were different in patients with PH versus controls. After adjusting for age, gender, and BMI, the serum NLRC3 concentrations were significantly lower in the PH patients than in the controls (1.49±1.07 ng/mL vs. control 4.44±2.29 ng/mL; p<0.05) (Fig. 1A and 1B).

Subsequently, we evaluated whether serum NLRC3 concentrations is different among PH patients grouped by WHOFC (FC II, n=6; FC III-IV, n=37). First, serum NLRC3 concentrations were significantly decreased in WHOFC II versus controls. Secondly, we detected further decreased NLRC3 expression in WHOFC III-IV compared to WHOFC II patients (Fig. 1C-D). There was no significant difference between WHO FC III and WHOFC IV (p>0.05).

**Figure 2.**
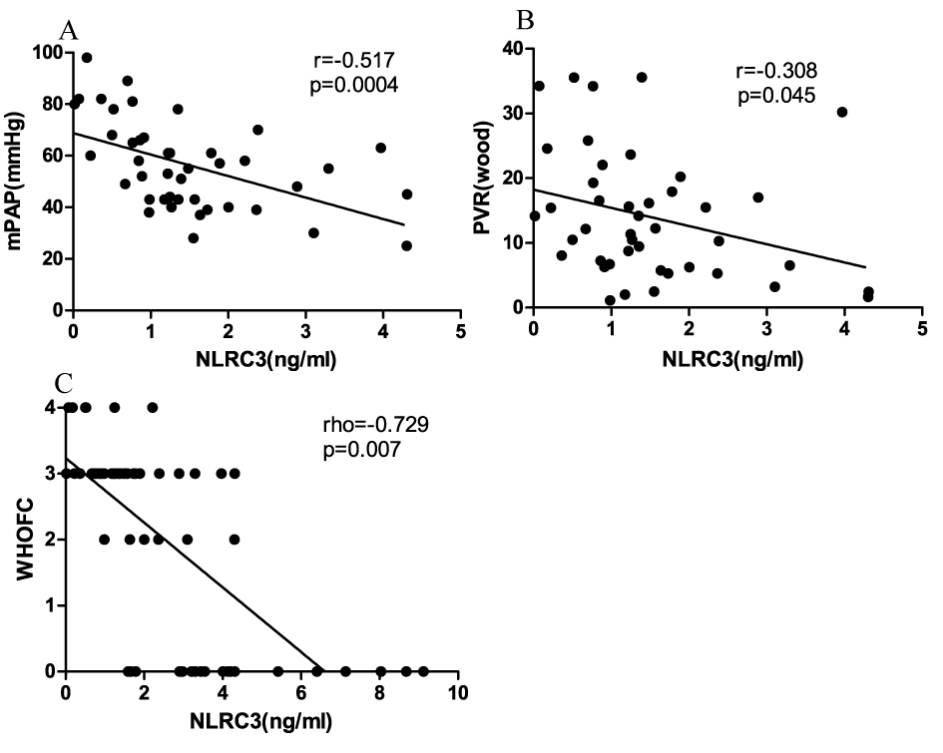
Serum NLRC3 concentrations negatively correlated with mPAP, PVR and WHOFC Serum NLRC3 concentrations significantly negatively correlated with mPAP (r = -0.517, p=0.0004, Fig. 2A), PVR (r = -0.308, p=0.045, Fig. 2B) and WHOFC (rho=-0.729, p=0.007, Fig. 2C) in PH patients.

### Serum NLRC3 concentrations were negatively correlated with mPAP and PVR obtained from RHC in PH patients

As RHC is the only way to certainly establish a hemodynamic diagnosis of PH [6], we analyzed the relevance between serum NLRC3 concentrations and hemodynamic parameters measured by RHC in PH patients. The data demonstrated that serum NLRC3 concentrations were negatively correlated with mPAP (r=-0.517, p=0.0004) and PVR (r=-0.308, p=0.045) in PH patients. However, there were no significant correlations among NLRC3 and PCWP, mRAP, CO and CI (Fig. 2A and 2B; Table 2). As we know, mPAP and PVR values were critical for determining progression of PH pathophysiology, and values from PCWP, mRAP, CO and CI were utilized in categorizing subtype of PH disease. No correlation was found between serum NLRC3 concentrations and echocardiography parameters.

Because of its non-invasive, commonly available and relatively affordable profiles, echocardiography is widely recommended to screen and monitor progression of PH [9]. Therefore, we examined the echocardiographic parameters of all selected subjects, including EF, TV, sPAP, PA, LA, LV, RA, and RV. The relationships between these markers and serum NLRC3 concentrations were studied. We did not find any correlations between serum NLRC3 concentrations and these parameters (p>0.05, Table 3).

### Serum NLRC3 concentrations negatively correlated with WHOFC in PH patients

Detection of exercise capacity is an integral part to assess the physical condition of PH patients. WHOFC is associated with outcomes in PH patients [14]. 6MWT and BDS can provide information about functional status, treatment efficacy and disease prognosis [15, 16, 24].

Our research found that there was a negative correlation between serum NLRC3 concentrations and WHOFC in PH patients (rho=-0.729, p=0.007, Fig. 2C); nevertheless the serum NLRC3 concentrations with 6MWT and BDS were not significantly correlated in PH patients (p>0.05, Table 4).

**Table 2:** Correlations between NLRC3 and hemodynamic parameters obtained by RHC in PH patients.

	NLRC3	
	r	*p*
mPAP	-0.517	0.0004*
mRAP	-0.119	0.448
PCWP	0.203	0.191
PVR	-0.308	0.045*
CO	0.298	0.053
CI	-0.0511	0.747

Serum NLRC3 concentrations were negatively correlated with mPAP (r=-0.517,* p*=0.0004) and PVR (r=-0.308, *p*=0.045) in PH patients. No significant correlations among NLRC3 and PCWP, mRAP, CO and CI. Abbreviations: mPAP: mean pulmonary artery pressure; mRAP: mean right atrial pressure; PCWP: pulmonary capillary wedge pressure; PVR: pulmonary vascular resistance; CO: cardiac output; CI: cardiac index.

* *p*<0.05

The cut-off value of Serum NLRC3 concentration was 2.90ng/mL to distinguish PH patients from healthy people.

**Figure 3.**
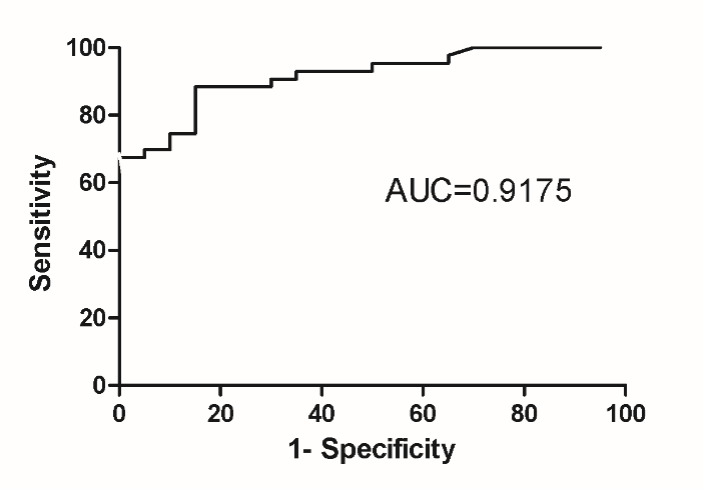
The cut-off value of Serum NLRC3 concentration was 2.90ng/mL to distinguish PH patients from healthy people The area under the curve was 0.9175 (95%CI 0.8487 to 0.9827), 2.897 ng/mL is the maximum cut-off value, at this point the sensitivity of PH was 88%, and the specificity was 85%

Since we discovered the difference of Serum NLRC3 concentration between PH and healthy control people, we intended to find the discriminate point to help diagnose PH. According to the serum NLRC3 concentrations of ROC curve, the results were statistically significant. The area under the curve was 0.9175 (95%CI 0.8487 to 0.9827), and 2.90ng/mL was the maximum cut-off value, at this point the sensitivity of PH was 88%, the specificity was 85% (Fig. 3).

**Table 3:** Correlations between NLRC3 and echocardiography parameters.

	NLRC3	
	r	*p*
TV	0.241	0217
sPAP	0.243	0.221
EF	0.374	0.096
PA/AO	0.008	0.969
PA	0.051	0.794
RA	0.261	0.179
RV	0.207	0.290
LA	0.145	0.527
LV	-0.152	0.440

There were no correlations between serum NLRC3 concentrations and echocardiography parameters (*p*>0.05). Abbreviations: TV: tricuspid regurgitation velocity; sPAP: pulmonary artery systolic pressure; PA: pulmonary artery diameter; EF: left ventricular ejection fraction; PA/AO: the pulmonary artery and aortic diameter; RA: apical four chamber view right atrium the transverse diameter; RV: apical four chamber view right ventricular diastolic diameter; LA: left ventricular long axis view of the left atrium diastolic diameter; LV: left ventricular long axis view of the left ventricular diastolic diameter.

## DISCUSSION

PH, which is characterized by a persistent elevation in mean pulmonary arterial pressure and pulmonary vascular remodeling, is a progressive disease that is often leading to right ventricular failure and death. PH is diagnosed when the mPAP exceeds 25mmHg at rest, as measured by RHC [25]. A 2013 epidemiological survey found that the prevalence of PH ranged from 5 to 15 cases per 1 million adults. The use of targeted drugs to a certain extent improve a patient’s condition, but the long-term prognosis of PH is still not optimistic [4]. Both early diagnosis and accurate stratification are required to improve the therapeutic strategies.

Studies on pulmonary hypertension diagnosis and treatment have increased over the past few decades. However, the pathogenesis of pulmonary hypertension is not yet fully defined. It is considered that many pathophysiological changes were consequences of multiple physiological pathways and cell phenotypes transformation, including pulmonary vasoconstriction, proliferation, vascular remodeling, inflammatory infiltration and in situ thrombosis. The role of immunity and inflammation has been increasingly noticed in PH[26-28]^.^ Numerous studies have demonstrated that inflammation and vascular remodeling are closely related [29, 30].

NLRs are a class of important pathogen-associated molecular pattern recognition receptors (PRRs) that are generally localized in the cytoplasm. They can recognize intracellular pathogens and dangerous signals to initiate innate immune responses [22, 31]. Most members of the NLRs family members contain three distinct functional domains: an N-terminal effect binding domain (EBD), a central nucleotide binding domain (NBD), and a leucine-rich repeats (LRRs) [32]. The majority of the NLRs family functions as inflammatory promoters. On the contrary, recent studies have found that NLRC3 might downregulate the production of pro-inflammatory cytokines and induction of pyroptosis by inhibiting the inflammasome [21, 33]. NLRC3 is located in the cytoplasm, which is composed of the EBD, the middle NACHT region and the LRRs at the C terminal. The EBD section from NLRC3 is different from EBD section of other NLRC family proteins, a difference which may contribute to its unique function in anti-inflammation and pyroptosis inhibition^[34]^. Schneider M and his members showed that NLRC3 attenuates Toll-like receptor signaling and NF-kappaB activation after lipopoly-saccharides exposure by interacting with TRAF6 [35]. Nowadays studies reveal that NLRC3 may negatively regulate inflammasome activity or other pathways mediating inflammatory signaling that involve apoptosis-associated speck-like protein because of the NACHT and unusual EBD structure [33, 36].

**Table 4:** Correlations between NLRC3 and functional indexes in PH patients.

	NLRC3	
	r/rho	*p*
WHOFC	-0.403	0.007*
6MWD	0.143	0.912
BDS	0.028	0.836

Serum NLRC3 concentrations negatively correlated with WHOFC in PH patients (rho=-0.729, *p=0.007); no significant correlations among serum NLRC3 concentrations and 6MWT, BDS in PH patients (p>0.05). Abbreviations: WHOFC: WHO pulmonary hypertension heart function classification; 6MWD: 6 minutes walking distance; BDS: Borg dyspnea score

It has been demonstrated that expression of NLRC3 is drastically reduced in the tumor tissues of patients with colorectal cancer compared to healthy tissues. Karki R and colleagues show that mice lacking NLRC3 are hyper-susceptible to colitis and colorectal tumorigenesis. Increased expression of NLRC3 protects against cellular proliferation and stem-cell-derived organoid formation. Decrease of NLRC3 contributes to the activation of pathways that induce inflammation, and promote cell proliferation, migration and extracellular matrix remodeling [23].

In case of NLRC3 gene mutation, the primitive macrophages spontaneously aggregate and increase pro-inflammatory cytokines thus initiate inflammatory response. These data suggest that NLRC3 may be able to prevent inappropriate inflammatory activation of macrophages [33]. Since macrophages are essential elements of the inflammatory infiltration in the lungs of PH patients [26, 37], we anticipate to observe changes of NLRC3 expression in PH patients.

In ourstudies, we found that serum NLRC3 concentrations were lower in PH patients than control subjects. We also discovered that serum NLRC3 concentrations were further decreased in severe PH patients. The serum levels of NLRC3 were significantly correlated with mPAP and PVR (P <0.05). As hemodynamic parameters especially PVR are the main feature and always represent the severity of PH[5], the correlation analysis indicates that NLRC3 is potentially an important index to reflect PH progression.. Up to the current time, echocardiography is still not considered to be the most accurate and sensitive method for PH diagnosis, this may explain the reason why no correlation detected between serum NLRC3 concentrations and echocardiography parameters.

Our data, for the first time, show that the degree of decreased serum NLRC3 in PH is associated with severity of WHO FC, thus suggesting NLRC3 may potentially be an indicator to evaluate disease severity. Since PH is characterized by serious angioproliferative vascular remodeling, decreased serum NLRC3 concentrations in PH may reflect vascular remodeling. However, we provide data from a single time point. Additional studies addressing the temporal courses of NLRC3 values and their possible correlation with development of PH disease are necessary.

WHOFC also indicated that NLRC3 was correlated with the severity of the disease, and longitudinal assessments of NLRC3 may be a good candidate for screening and monitoring disease progression. Due to the small sample size, there were no significantly differences detected between NLRC3 and other indexes, such as 6MWD. The ROC curve analysis revealed that NLRC3 appeared a high sensitivity and specificity at the concentration of 2.897 ng/mL.

Taken together, the present study presents evidence that increased mPAP and PVR in PH were associated with decreased NLRC3 concentrations. We provide evidence for NLRC3 in the process of human PH, and suggest the potential application as a new biomarker in clinical PH. This study provides the rational for further clinical and experimental studies investigating the NLRC3 in the pathogenesis of PH. However, as the specimens were limited to peripheral blood, it can only explain that NLRC3 may be associated with PH. The role of NLRC3 in the mechanism of PH still needs further research. In addition, due to the limited observation time, our study lacks to provide prognosis and survival data. Future studiesshould add follow-up monitoring while expanding sample size. The mechanisms of NLRC3 involved in PH also need further research.

### Conclusions

Serum NLRC3 concentration is significantly decreased in PH patients, and is negatively associated with the severity of the disease. Measurement of serum NLRC3 concentration may help early diagnosis and monitoring progression of PH, thus guide accurate clinical decision-making and appropriate therapeutic interventions in PH patients. However, further mechanistic study is needed to fully explore the roles of NLRC3 in PH.

## References

[1] Nicolls MR, Voelkel NF (2017). The Roles of Immunity in the Prevention and Evolution of Pulmonary Arterial Hypertension. Am J Resp Crit Care, 195(10):1292-9. 10.1164/rccm.201608-1630PP PMC5443903 27786553

[2] Humbert M, Sitbon O, Chaouat A, Bertocchi M, Habib G, Gressin V, et al (2010). Survival in patients with idiopathic, familial, and anorexigen-associated pulmonary arterial hypertension in the modern management era. Circulation, 122(2):156-63. 20585011 10.1161/CIRCULATIONAHA.109.911818

[3] Galie N, Humbert M, Vachiery JL, Gibbs S, Lang I, Torbicki A, et al (2016). 2015 ESC/ERS Guidelines for the diagnosis and treatment of pulmonary hypertension: The Joint Task Force for the Diagnosis and Treatment of Pulmonary Hypertension of the European Society of Cardiology (ESC) and the European Respiratory Society (ERS): Endorsed by: Association for European Paediatric and Congenital Cardiology (AEPC), International Society for Heart and Lung Transplantation (ISHLT). Eur Heart J, 37(1):67-119. 26320113 10.1093/eurheartj/ehv317

[4] Rich JD, Rich S (2014). Clinical diagnosis of pulmonary hypertension. Circulation, 130(20):1820-30. 25385937 10.1161/CIRCULATIONAHA.114.006971

[5] Galie N, Humbert M, Vachiery JL, Gibbs S, Lang I, Torbicki A, et al (2015). 2015 ESC/ERS Guidelines for the diagnosis and treatment of pulmonary hypertension: The Joint Task Force for the Diagnosis and Treatment of Pulmonary Hypertension of the European Society of Cardiology (ESC) and the European Respiratory Society (ERS): Endorsed by: Association for European Paediatric and Congenital Cardiology (AEPC), International Society for Heart and Lung Transplantation (ISHLT). Eur Respir J, 46(4):903-75. 26318161 10.1183/13993003.01032-2015

[6] Hoeper MM, Lee SH, Voswinckel R, Palazzini M, Jais X, Marinelli A, et al (2006). Complications of right heart catheterization procedures in patients with pulmonary hypertension in experienced centers. J Am Coll Cardiol, 48(12):2546-52. 17174196 10.1016/j.jacc.2006.07.061

[7] Rosenkranz S, Preston IR (2015). Right heart catheterisation: best practice and pitfalls in pulmonary hypertension. Eur Respir Rev, 24(138):642-52. 26621978 10.1183/16000617.0062-2015 PMC9487613

[8] Fisher MR, Forfia PR, Chamera E, Housten-Harris T, Champion HC, Girgis RE, et al (2009). Accuracy of Doppler echocardiography in the hemodynamic assessment of pulmonary hypertension. Am J Resp Crit Care, 179(7):615-21. 10.1164/rccm.200811-1691OC PMC2720125 19164700

[9] Janda S, Shahidi N, Gin K, Swiston J (2011). Diagnostic accuracy of echocardiography for pulmonary hypertension: a systematic review and meta-analysis. Heart, 97(8):612-22. 21357375 10.1136/hrt.2010.212084

[10] Rich JD, Shah SJ, Swamy RS, Kamp A, Rich S (2011). Inaccuracy of Doppler echocardiographic estimates of pulmonary artery pressures in patients with pulmonary hypertension: implications for clinical practice. Chest, 139(5):988-93. 20864617 10.1378/chest.10-1269

[11] Finkelhor RS, Lewis SA, Pillai D (2015). Limitations and strengths of doppler/echo pulmonary artery systolic pressure-right heart catheterization correlations: a systematic literature review. Echocardiography, 32(1):10-8. 24661140 10.1111/echo.12594

[12] Bradlow WM, Gibbs JS, Mohiaddin RH (2012). Cardiovascular magnetic resonance in pulmonary hypertension. J Cardiov Magn Reson, 14:6. 10.1186/1532-429X-14-6 PMC3305675 22257586

[13] Galie N, Hoeper MM, Humbert M, Torbicki A, Vachiery JL, Barbera JA, et al (2009). Guidelines for the diagnosis and treatment of pulmonary hypertension: the Task Force for the Diagnosis and Treatment of Pulmonary Hypertension of the European Society of Cardiology (ESC) and the European Respiratory Society (ERS), endorsed by the International Society of Heart and Lung Transplantation (ISHLT). Eur Heart J, 30(20):2493-537. 19713419 10.1093/eurheartj/ehp297

[14] Barst RJ, Chung L, Zamanian RT, Turner M, McGoon MD (2013). Functional class improvement and 3-year survival outcomes in patients with pulmonary arterial hypertension in the REVEAL Registry. Chest, 144(1):160-8. 23429998 10.1378/chest.12-2417

[15] Khair RM, Nwaneri C, Damico RL, Kolb T, Hassoun PM, Mathai SC (2016). The Minimal Important Difference in Borg Dyspnea Score in Pulmonary Arterial Hypertension. Ann Am Thorac Soc, 13(6):842-9. 26974862 10.1513/AnnalsATS.201512-824OC PMC5018926

[16] Salzman SH (2009). The 6-min walk test: clinical and research role, technique, coding, and reimbursement. Chest, 135(5):1345-52. 19420202 10.1378/chest.07-1682

[17] Fenster BE, Lasalvia L, Schroeder JD, Smyser J, Silveira LJ, Buckner JK, et al (2016). Galectin-3 levels are associated with right ventricular functional and morphologic changes in pulmonary arterial hypertension. Heart Vessels, 31(6):939-46. 25976729 10.1007/s00380-015-0691-z

[18] Calvier L, Legchenko E, Grimm L, Sallmon H, Hatch A, Plouffe BD, et al (2016). Galectin-3 and aldosterone as potential tandem biomarkers in pulmonary arterial hypertension. Heart, 102(5):390-6. 26869635 10.1136/heartjnl-2015-308365

[19] Nagaya N, Nishikimi T, Uematsu M, Satoh T, Kyotani S, Sakamaki F, et al (2000). Plasma brain natriuretic peptide as a prognostic indicator in patients with primary pulmonary hypertension. Circulation, 102(8):865-70. 10952954 10.1161/01.cir.102.8.865

[20] Suzuki S, Yoshihisa A, Yokokawa T, Misaka T, Sakamoto N, Sugimoto K, et al (2016). Association between levels of anti-angiogenic isoform of vascular endothelial growth factor A and pulmonary hypertension. Int J Cardiol, 222:416-20. 27505326 10.1016/j.ijcard.2016.07.277

[21] Davis BK, Wen H, Ting JP (2011). The inflammasome NLRs in immunity, inflammation, and associated diseases. Annu Rev Immuno, 29:707-35. 10.1146/annurev-immunol-031210-101405 PMC4067317 21219188

[22] Chen G, Shaw MH, Kim YG, Nunez G (2009). NOD-like receptors: role in innate immunity and inflammatory disease. Annu Rev Pathol, 4:365-98. 18928408 10.1146/annurev.pathol.4.110807.092239

[23] Karki R, Man SM, Malireddi RK, Kesavardhana S, Zhu Q, Burton AR, et al (2016). NLRC3 is an inhibitory sensor of PI3K-mTOR pathways in cancer. Nature, 540:583-7 10.1038/nature20597 PMC5468516 27951586

[24] Rasekaba T, Lee AL, Naughton MT, Williams TJ, Holland AE (2009). The six-minute walk test: a useful metric for the cardiopulmonary patient. Intern Med J, 39(8):495-501. 19732197 10.1111/j.1445-5994.2008.01880.x

[25] Hoeper MM, Bogaard HJ, Condliffe R, Frantz R, Khanna D, Kurzyna M, et al (2013). Definitions and diagnosis of pulmonary hypertension. J Am Coll Cardiol, 62(25 Suppl):D42-50. 24355641 10.1016/j.jacc.2013.10.032

[26] Tian W, Jiang X, Tamosiuniene R, Sung YK, Qian J, Dhillon G, et al (2013). Blocking macrophage leukotriene b4 prevents endothelial injury and reverses pulmonary hypertension. Sci Transl Med, 5(200):200ra117. 10.1126/scitranslmed.3006674 PMC4016764 23986401

[27] Nicolls MR, Voelkel NF (2016). The Roles of Immunity in the Prevention and Evolution of Pulmonary Arterial Hypertension. Am J Resp Crit Care,195(10):1292-9 10.1164/rccm.201608-1630PP PMC5443903 27786553

[28] Nicolls MR, Taraseviciene-Stewart L, Rai PR, Badesch DB, Voelkel NF (2005). Autoimmunity and pulmonary hypertension: a perspective. Eur Respir J, 26(6):1110-8. 16319344 10.1183/09031936.05.00045705

[29] Vaillancourt M, Ruffenach G, Meloche J, Bonnet S (2015). Adaptation and remodelling of the pulmonary circulation in pulmonary hypertension. Can J Cardiol, 31(4):407-15. 25630876 10.1016/j.cjca.2014.10.023

[30] Pugliese SC, Poth JM, Fini MA, Olschewski A, El Kasmi KC, Stenmark KR (2015). The role of inflammation in hypoxic pulmonary hypertension: from cellular mechanisms to clinical phenotypes. Am J Physiol Lung Cell Mol, 308(3):L229-52. 10.1152/ajplung.00238.2014 PMC4338929 25416383

[31] Mason DR, Beck PL, Muruve DA (2012). Nucleotide-binding oligomerization domain-like receptors and inflammasomes in the pathogenesis of non-microbial inflammation and diseases. J Innate Immun, 4(1):16-30. 22067846 10.1159/000334247

[32] Ting JP, Davis BK (2005). CATERPILLER: a novel gene family important in immunity, cell death, and diseases. Annu Rev Immunol, 23:387-414. 15771576 10.1146/annurev.immunol.23.021704.115616

[33] Shiau CE, Monk KR, Joo W, Talbot WS (2013). An anti-inflammatory NOD-like receptor is required for microglia development. Cell Rep, 5(5):1342-52. 24316075 10.1016/j.celrep.2013.11.004 PMC3878655

[34] Harton JA, Linhoff MW, Zhang J, Ting JP (2002). Cutting edge: CATERPILLER: a large family of mammalian genes containing CARD, pyrin, nucleotide-binding, and leucine-rich repeat domains. J Immunol, 169(8):4088-93. 12370334 10.4049/jimmunol.169.8.4088

[35] Schneider M, Zimmermann AG, Roberts RA, Zhang L, Swanson KV, Wen H, et al (2012). The innate immune sensor NLRC3 attenuates Toll-like receptor signaling via modification of the signaling adaptor TRAF6 and transcription factor NF-kappaB. Nat Immunol, 13(9):823-31. 22863753 10.1038/ni.2378 PMC3721195

[36] Sarkar A, Duncan M, Hart J, Hertlein E, Guttridge DC, Wewers MD (2006). ASC directs NF-kappaB activation by regulating receptor interacting protein-2 (RIP2) caspase-1 interactions. J Immunol, 176(8):4979-86. 16585594 10.4049/jimmunol.176.8.4979

[37] Tamosiuniene R, Tian W, Dhillon G, Wang L, Sung YK, Gera L, et al (2011). Regulatory T cells limit vascular endothelial injury and prevent pulmonary hypertension. Circ Res, 109(8):867-79. 21868697 10.1161/CIRCRESAHA.110.236927 PMC3204361

